# Prospective Prediction of Dapaconazole Clinical Drug–Drug Interactions Using an In Vitro to In Vivo Extrapolation Equation and PBPK Modeling

**DOI:** 10.3390/ph16010028

**Published:** 2022-12-26

**Authors:** Natalícia de Jesus Antunes, Fernanda de Lima Moreira, Karin Kipper, Lewis Couchman, Daniel Temponi Lebre, Atholl Johnston, Gilberto De Nucci

**Affiliations:** 1 Department of Pharmacology, Faculty of Medical Sciences, State University of Campinas (UNICAMP), Campinas 13083-881, SP, Brazil; 2 Faculty of Pharmacy, Federal University of Rio de Janeiro, Rio de Janeiro 21941-902, RJ, Brazil; 3 Institute of Chemistry, University of Tartu, 14a Ravila Street, 50411 Tartu, Estonia; 4 Analytical Services International (ASI) Ltd., St George’s—University of London, Cranmer Terrace, London SW17 0RE, UK; 5 Pharmaceutical Sciences Clinical Academic Group, King’s College London, London SE1 9NH, UK; 6 Center for Applied Mass Spectrometry (CEMSA), São Paulo 05508-000, SP, Brazil; 7 Clinical Pharmacology, William Harvey Research Institute, Barts and The London School of Medicine and Dentistry, Queen Mary University of London, London EC1M 6BQ, UK; 8 Institute of Biomedical Sciences, University of São Paulo (USP), São Paulo 05508-000, SP, Brazil; 9 Faculty of Medicine, São Leopoldo Mandic (SLMANDIC), Campinas 13045-755, SP, Brazil

**Keywords:** dapaconazole, cytochrome P450, inhibition, in vitro evaluation, human liver microsomes, IVIVE, PBPK model

## Abstract

This study predicted dapaconazole clinical drug–drug interactions (DDIs) over the main Cytochrome P450 (CYP) isoenzymes using static (in vitro to in vivo extrapolation equation, IVIVE) and dynamic (PBPK model) approaches. The in vitro inhibition of main CYP450 isoenzymes by dapaconazole in a human liver microsome incubation medium was evaluated. A dapaconazole PBPK model (Simcyp version 20) in dogs was developed and qualified using observed data and was scaled up for humans. Static and dynamic models to predict DDIs following current FDA guidelines were applied. The in vitro dapaconazole inhibition was observed for all isoforms investigated, including CYP1A2 (IC_50_ of 3.68 µM), CYP2A6 (20.7 µM), 2C8 (104.1 µM), 2C9 (0.22 µM), 2C19 (0.05 µM), 2D6 (0.87 µM), and 3A4 (0.008–0.03 µM). The dynamic (PBPK) and static DDI mechanistic model-based analyses suggest that dapaconazole is a weak inhibitor (AUCR > 1.25 and <2) of CYP1A2 and CYP2C9, a moderate inhibitor (AUCR > 2 and <5) of CYP2C8 and CYP2D6, and a strong inhibitor (AUCR ≥ 5) of CYP2C19 and CYP3A, considering a clinical scenario. The results presented may be a useful guide for future in vivo and clinical dapaconazole studies.

## 1. Introduction

Dapaconazole, a novel imidazole, has shown antifungal activity against several pathogenic fungi, such as *Tricophyton verrucosum*, *Tricophyton rubrum*, *Trycophyton mantagrophutes*, *Microsporum gypseum*, *Microsporum canis*, and *Aspergillus niger* [1,2,3,4,5,6,7]. Dapaconazole demonstrated noninferior efficacy to miconazole for the topical treatment of tinea cruris [4], to ketoconazole for treating interdigital tinea pedis [3], and pityriasis versicolor [5]. In addition, dapaconazole has shown a good safety profile in all trials.

Current concern about the use of azole antifungals in clinical practice is the many drug–drug interactions (DDIs) related to these drugs, mainly as moderate/strong inhibitors of cytochrome P450 (CYP) isoenzymes [8,9]. The investigation of a potential DDI of a new azole antifungal in the early drug development process is critical to allow the safe use of the new molecular entity during clinical trials and later during clinical practice [10]. Therefore, understanding and identifying the enzymes that are inhibited in the presence of the drug under study is directly related to deciding whether to proceed or not in the development phase [11]. Mechanistic approaches such as static (in vitro to in vivo extrapolation equations, IVIVE) or dynamic models (physiologically-based pharmacokinetic (PBPK) models) incorporating in vitro data of human systems are being used increasingly to predict the clinical DDI potential associated with new chemical entities to help in the drug development process [12,13].

This work aimed to predict dapaconazole clinical DDIs over the main CYP450 isoenzymes using static (IVIVE) and dynamic (PBPK model) approaches.

## 2. Results

### 2.1. In Vitro DDI

Table 1 summarizes the conditions of the in vitro CYP450 metabolism inhibition assay. 

The LC-MS/MS conditions are described in Table 2.

The IC_50_ values of the dapaconazole inhibition of CYP450 isoforms obtained in vitro are shown in Table 3. Inhibition was observed for all isoforms investigated, CYP1A2 (3.68 µM), CYP2A6 (20.7 µM), 2C8 (104.1 µM), 2C9 (0.22 µM), 2C19 (0.05 µM), 2D6 (0.87 µM), and 3A4 (0.008–0.03 µM). The positive inhibitors furafylline (CYP1A2), tranylcypromine (CYP2A6 and CYP2C19), quercetin (CYP2C8), sulfaphenazole (CYP2C9), quinidine (CYP2D6), and ketoconazole (CYP3A) have been shown to be potent inhibitors.

### 2.2. Development of PBPK Model in Dogs

A PBPK model was first developed in dogs using a hybrid PBPK approach employing the in vivo clearance reported in dogs [7]. The input parameters are described in Table 4.

**Figure 1:**
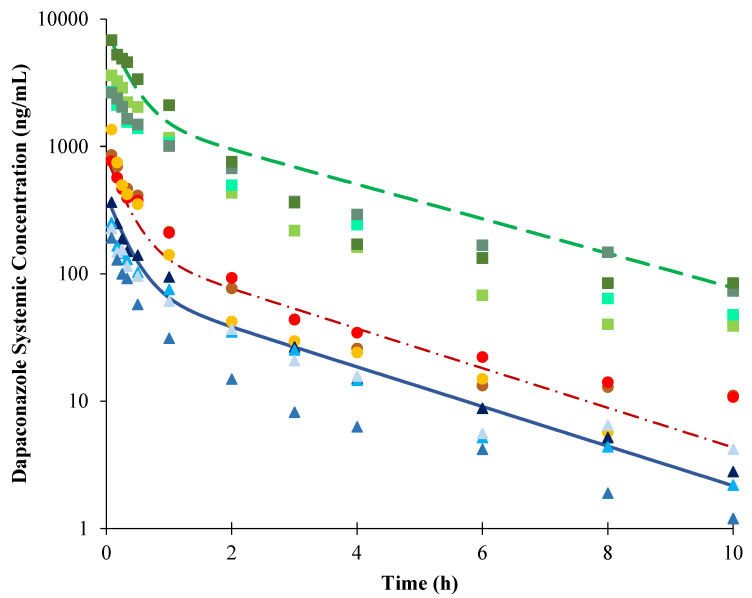
Plasma concentrations versus time plots for dapaconazole after intravenous dose administration in dogs. The markers represent the observed data in dogs from Palo et al. [7], and the lines represent the predicted mean concentration versus time profiles obtained with the PBPK model. The solid line and triangle markers represent predicted and observed plasma concentrations after a 1 mg/kg dose; the dash-dot line and circle markers represent predicted and observed plasma concentrations after a 2 mg/kg dose; and the dashed line and square markers represent predicted and observed plasma concentrations after a 20-mg/kg dose.

First, we used the raw data from Palo et al. [7] to obtain the dapaconazole compartmental model employing Phoenix^®^ WinNonlin^®^, version 6.3 (Certara). The intravenous dapaconazole exposure was described as a bicompartmental model (data not shown). The PBPK model was first developed using a single 2 mg/kg intravenous dose of dapaconazole and employing the reported in vivo clearance of 591.7 L/min [7] in a hybrid approach. Then, the full PBPK model, which used the method 2 (Rodgers and Rowland [15]) to predict the volume of distribution at steady-state conditions (V_ss_) was evaluated. The sensitivity analysis estimated the tissue-to-plasma partition coefficient (K_p_) value of 0.01 to best fit the observed values. Despite that, the full PBPK model did not describe the dapaconazole bicompartmental profile. Considering this issue, in addition to the low volume of distribution (V_d_) observed in dogs, the minimal PBPK plus a single adjusting compartment (SAC) was selected. The V_sac_ (apparent volume of SAC), k_in_ (rate constant from systemic compartment to SAC), and k_out_ (rate constant from SAC compartment to the systemic compartment) values were determined from the parameter estimation approach selecting the values of 3.88 L/kg, 0.026 h^−1^, and 0.016 h^−1^, respectively, that better described the observed PK profile. The predicted V_ss_ using Rodgers and Rowland’s method [15] was 6.36 L/kg. The developed PBPK model described the observed dapaconazole PK profile in dogs reasonably well after intravenous single doses of 1 mg/kg, 2 mg/kg, or 20 mg/kg (Figure 1); furthermore, the PK parameters’ observed/predicted ratios were between 0.5-fold to 2-fold (Table 5).

To predict the role of CYP450 liver enzymes on the elimination of dapaconazole in dogs, the intrinsic clearance value obtained from dog liver microsomes (257.97 µL/min/mg) [14] was incorporated as the single elimination pathway in dogs. The predicted systemic clearance obtained after a single intravenous dose of 20 mg/kg dapaconazole from the model incorporating intrinsic clearance from dog liver microsomes in an in vitro model (164 mL/min) was 3.3-fold lower than the predicted systemic clearance (454.7 mL/min) from the model incorporating in vivo clearance in dogs. Considering this, the in vivo clearance value in dogs, and not intrinsic clearance from the microsome, was applied in the allometric scaling formula to predict clearance in humans.

### 2.3. Extrapolation of the PBPK Model Developed in Dogs to Humans

The allometry tools provided by Simcyp were employed to extrapolate the V_ss_ and clearance (CL) from dogs to humans. Simple allometry with one species (dog) considered V_ss, human_ = V_ss,animal_ (L/kg) [16], and CL_human_ = CL_animal_ (mL/day) [17]. The input parameters included in the dapaconazole PBPK model for humans are described in Table 4.

To predict the role of CYP450 liver enzymes in the elimination of dapaconazole in humans, the intrinsic clearance value obtained from human liver microsomes (118.5 µL/min/mg) [14] was incorporated as the single elimination pathway in humans.

The elimination half-life (t_1/2_) observed after simulating a single 20 mg/kg intravenous dose of (in an adult of 73 kg corresponding to a 1.460-mg dose) dapaconazole was 7.9 h. As an exercise in the simulation of potential DDI, we selected an intravenous dose of 500 mg every 8 h as a dosing regimen.

### 2.4. DDI Prediction of Dapaconazole as an Inhibitor

First, dapaconazole inhibition potential was evaluated by calculating the R1 value for reversible inhibition, demonstrating that dapaconazole has clinical inhibition potential in all CYP isoforms tested, except CYP2C8. To assess the clinical potential interaction of dapaconazole to the exposure (area under the curve ratio—AUC) of CYP substrate drugs, dapaconazole was evaluated as an inhibitor using static and dynamic models to predict DDI in clinical scenarios (Table 6) [18]. Considering both results from static and dynamic models, the worst-case scenario (higher AUCR value) was considered to classify the potential of dapaconazole as an inhibitor according to FDA classification [13]. Dapaconazole is a potential clinically weak inhibitor of CYP1A2 and CYP2C9, a moderate inhibitor of CYP2D6 and CYP2C8, and a strong inhibitor of CYP2C19 and CYP3A4.

## 3. Discussion

Azole antifungals are used as a primary treatment for fungal infections, and to support the treatment of immune-suppressed patients, for example, organ transplant patients and patients with acquired immunodeficiency syndrome, who also use other medications. Azole antifungals can change the exposure of these medications due to DDIs caused by inhibiting drug-metabolizing enzymes [9,21]. Drug interactions represent a major problem in drug therapy. Therefore, prior knowledge of these interactions during the development process of new drugs through in vitro enzyme inhibition studies is of great value, as this helps in avoiding adverse reactions generated by the interactions [22].

Dapaconazole is metabolized by CYP450 enzymes in the liver as previously demonstrated by our research group using in vitro studies with liver microsomes from humans, dogs, and rats [14]. Other azole antifungals such as itraconazole and voriconazole also are substrates of CYP450 enzymes. In addition, azole antifungals are well-known CYP450 inhibitors, and the inhibition of CYP3A4 enzymatic activity is considered the main source of DDIs by these drugs [23,24,25,26,27].

In liver microsomes, the inhibitory potency of a drug can be measured by determining the inhibition constant required to achieve half the maximum inactivation rate for reversible inhibition (Ki) or IC_50_ of a specific substrate for each CYP isoform. IC_50_ values are typically classified into low (IC_50_ > 10 μM), moderate (1 μM < IC_50_ < 10 μM), and high (IC_50_ < 1 μM) reversible inhibition [11]. Considering these values, dapaconazole in vitro slightly inhibits CYP2A6 and CYP2C8, moderately inhibits CYP1A2, and highly inhibits CYP2C9, CYP2C19, CYP2D6, and CYP3A. Comparing dapaconazole with other azole antifungals evaluated in vitro, fluconazole moderately inhibits CYP2C9 and CYP3A; itraconazole strongly inhibits CYP2B6 and CYP3A; ketoconazole strongly inhibits CYP1A1 and CYP3A and moderately inhibits CYP1A2, CYP2A6, and CYP2C9; miconazole strongly inhibits CYP2A6, CYP2B6, CYP2C9, CYP2C19, CYP2D6, and CYP3A; posaconazole strongly inhibits CYP3A; and voriconazole strongly inhibits CYP2B6 and CYP3A, and moderately inhibits CYP2C9 and CYP2C19 [9].

Considering that one important application of the PBPK model during early drug development is to predict drug exposure prior to in vivo studies (mainly clinical trials), we employed this valuable tool to build a dapaconazole PBPK model in dogs, and recently it was extrapolated to humans in order to predict potential DDI scenarios in humans. The PBPK model developed in dogs, using a middle-out approach considering the observed in vivo clearance previously reported by Palo et al. [7], was qualified with the observed data through a visual predictive check and 0.5- to 2-fold ratio difference observed to predict the pharmacokinetic parameter value (Figure 1 and Table 5).

After developing and qualifying the PBPK model in dogs, we extrapolated the model to humans, assuming the same CL and V_ss_ values from dogs. Other input parameters for the dapaconazole PBPK model in humans are described in Table 4. Later, we extrapolated this PBPK model to humans to perform a prospective clinical DDI prediction obtaining the AUCR dynamic (Table 6).

The DDI potential analysis from Ki and R1 values (Table 6) indicates that dapaconazole has a low potential to inhibit CYP2C8 (Ki: 52.1 µM; R1: 1.01), while it has a moderate potential to inhibit CYP1A2 (Ki: 1.84 µM; R1: 1.20) and high potential to inhibit CYP2C9 (Ki: 0.11 µM; R1: 4.43), CYP2C19 (Ki: 0.03 µM; R1: 15.14), CYP2D6 (Ki: 0.43 µM; R1: 1.86), and CYP3A (Ki: 0.004 µM; R1: 98.26 for midazolam substrate; Ki: 0.015 µM; R1: 25.70 for nifedipine substrate).

The results of the DDI static and dynamic mechanistic models indicated that dapaconazole is a weak inhibitor of CYP1A2 and CYP2C9, a moderate inhibitor of CYP2C8 and CYP2D6, and a strong inhibitor of CYP2C19 and CYP3A, according to FDA guidelines [13]. In general, the AUCR static and dynamic values for each CYP isoform evaluated correlated reasonably well. To critically evaluate these results, it is important to highlight that the static model considers the worst-case scenario, assuming that the inhibitor concentration is maintained at the maximum plasma concentration (C_max_) throughout the entire timed course, while the dynamic model uses the concentration versus time profiles of both inhibitor and substrate drugs, giving a more realistic prediction of a clinical DDI. Considering the AUCR ≥ 1.25 values obtained, we indicate that a clinical DDI study using a sensitive index substrate should be further performed for all CYP isoforms evaluated in the current work.

Clinical DDI studies demonstrated that fluconazole, itraconazole, ketoconazole, posaconazole, and voriconazole are moderate to strong CYP3A inhibitors, and fluconazole, itraconazole, ketoconazole, miconazole, and voriconazole are moderate CYP2C9 inhibitors [9]. The findings of the present study indicate that dapaconazole has the same characteristics as other azoles as an inhibitor of many CYP isoforms with a moderate to strong inhibition in CYP3A, but a weak inhibition in CYP2C9.

## 4. Materials and Methods

### 4.1. Chemicals and Reagents

Dapaconazole was obtained from Biolab Sanus Farmacêutica Ltd.a. (São Paulo, Brazil). Acetaminophen, bufuralol, coumarin, dextrorphan, diazepam, 4′-hydroxydiclofenac, ketoconazole, midazolam, 1-hydroxymidazolam, paclitaxel, phenacetin, sulindac, and formic acid (99% to 100%) were obtained from Sigma-Aldrich (St Louis, MO, USA). Nifedipine and dehydronifedipine were purchased from Cayman Chemical (Ann Arbor, MI, USA). Bupropion, clopidogrel, quinidine, sulfaphenazole, 4′-hydroxymephenytoin, 7-hydroxycoumarin, hydroxybupropion, 6α-hydroxypaclitaxel, and 1′-hydroxybufuralol, were purchased from Toronto Research Chemicals (Toronto, ON, Canada). Diclofenac, furafylline, S-mephenytoin, quercetin, and tranylcypromine were purchased from Santa Cruz Biotechnology (Dallas, TX, USA). The HPLC-grade solvents acetonitrile, ethyl acetate, methyl tert-butyl ether, chloroform, and methanol were obtained from Rathburn Chemicals (Walkerburn, Scotland). Aqueous phosphate buffer (0.5 mol/L, pH 7.4), reduced nicotinamide adenine dinucleotide phosphate (NADPH) regenerating system solutions A and B, and pooled HLMs (150 donors pool), RLMs (male pool), and BLMs (male pool) were purchased from Corning (Woburn, MA, USA). Deionized water was obtained in-house using a Synergy UV^®^ purification system (Millipore, Molsheim, France).

The standard stock solutions of the probe substrates were prepared in methanol at the following concentrations: phenacetin 5 mg/mL, coumarin 2 mg/mL, bupropion 5 mg/mL, paclitaxel 1 mg/mL, diclofenac 2 mg/mL, S-mephenytoin 5 mg/mL, bufuralol 1 mg/mL, midazolam 100 μg/mL, and nifedipine 1 mg/mL. The standard stock solutions of the markers (acetaminophen, 7-hydroxycoumarin, hydroxybupropion, 6α-hydroxypaclitaxel, 4′-hydroxydiclofenac, 4′-hydroxymephenytoin, 1′-hydroxybufuralol, 1′-hydroxymidazolam, and dehydronifedipine) were prepared in methanol at 1mg/mL. The standard stock solutions of the inhibitors were prepared in methanol at the following concentrations: furafylline 5 mg/mL, tranylcypromine 1 mg/mL, clopidogrel 1 mg/mL, quercetin 5 mg/mL, sulfaphenazole 1 mg/mL, quinidine 2 mg/mL, and ketoconazole (1 mg/mL). The standard stock solutions of the internal standards (IS) were prepared in methanol at the following concentrations: sulindac 1 mg/mL, dextrorphan 5 mg/mL, and diazepam 1 mg/mL. All solutions were stored in amber vials at −20 °C.

### 4.2. In Vitro DDI

To identify the potential of dapaconazole to inhibit CYP450, the activity of CYP1A2, CYP1A6, CYP2C8, CYP2C9, CYP2C19, CYP2D6, and CYP3A4/5 was evaluated in HLM using selective substrates in concentrations near to their Km and inhibitors (positive control) of CYP450, as shown in Table 1. The incubation conditions employed for each CYP450 isoform were performed, as previously described [28,29,30,31,32,33,34,35].

Solutions (n = 3) containing 10 μL of probe substrate of each CYP450 isoform with or without 10 µL dapaconazole (0.01, 0.1, 1, 10, and 100 µM) or specific inhibitors were added to 1.5 mL propylene tubes and evaporated to dryness. Buffer solution (0.1 mol/L aqueous phosphate buffer, pH 7.4, 69 µL), NADPH-regenerating solutions (10 µL solution A: 26 mmol/L NADP+, 66 mmol/L glucose-6-phosphate, and 66 mmol/L MgCl_2_; 2 µL solution B: 40 U/mL glucose-6-phosphate dehydrogenase in 5 mmol/L sodium citrate), and deionized water (69 µL) were added, in a total volume of 150 μL. The tubes were vortex-mixed and preincubated with continuous gentle shaking for 5 min at 37 °C in a shaking water bath. Reactions were initiated by the addition of 50 µL of HLM solution in the tubes which were gently mixed by hand and incubated with continuous gentle shaking at 37 °C. Aliquots of 200 µL ice-cold solvent (Table 1) containing IS (1 µg/mL) were added. The samples were vortexed for 5 min at 2000 rpm in a VXR basic Vibrax^®^ (Staufen, Germany) and then centrifuged at 16,000× *g* (Hettich^®^ MIKRO 185, Tuttlingen, Germany) for 15 min at 25 °C. The supernatants were transferred to glass autosampler vials and submitted to LC-MS/MS analysis (5 µL injection volume) to monitor the substrate metabolite formation.

### 4.3. Analysis by LC-MS/MS

The liquid chromatography (HPLC) system (Agilent Technologies, Santa Clara, CA, USA) consisted of a 1290 binary LC pump, a 1290 Infinity II Series^TM^ autosampler, and an MCT 1290 column oven. Dapaconazole was separated in a Luna^TM^ Omega polar C18 column (150 × 2.1 mm, 5-µm particle size; Phenomenex, Torrance, CA, USA) held at 40 °C using deionized water + 0.1% formic acid as mobile phase A and acetonitrile + 0.1% formic acid as mobile phase A at a flow rate of 0.3 mL/min. The applied gradient program consisted of 10% B, followed by a linear change to 100% B over 3 min. Mobile phase percentage B was then kept at 100% for 1 min and returned to initial conditions over 0.2 min (total run time of 5 min). The temperature of the autosampler was maintained at 5 °C.

Analytes and ISs were monitored in an API 4000™ triple quadrupole mass-spectrometer (AB Sciex, Concord, ON, Canada) with positive heated ion spray (Positive TurboIonSpray, MH^+^) for analyte detection. Source conditions were gas (high-purity nitrogen) temperature of 300 °C, collision gas of 3 mTorr, and IonSpray voltage of 5000 V. The analysis was performed in multiple reaction monitoring (MRM) mode. The MRM transitions, collision energy (CE), and collision cell exit potential (CXP) are presented in Table 2. The data acquisition and quantification were performed using Analyst^TM^ version 1.3.2 (AB Sciex, Concord, ON, Canada).

### 4.4. IC_50_ Determination

The remaining CYP450 activity was calculated by comparing samples in the presence and absence of dapaconazole or selective inhibitors, according to Equation (1):

(1) $$\%\mathrm{REA}=\frac{A_{i}}{A_{0\;}\cdot \;100}$$

where %REA is the percentage of remaining enzymatic activity, Ai corresponds to the metabolite-to-IS peak area ratio in the presence of dapaconazole or selective inhibitors, and A_0_ corresponds to the metabolite-to-IS peak area ratio in the absence of dapaconazole or selective inhibitors.

IC_50_ values were determined by a nonlinear regression of the %REA of each CYP450 isoform versus the logarithm of inhibitor concentration, using GraphPad Prism version 5.01 software (GraphPad Software, San Diego, CA, USA).

### 4.5. PBPK Model Strategy

**Figure 2:**
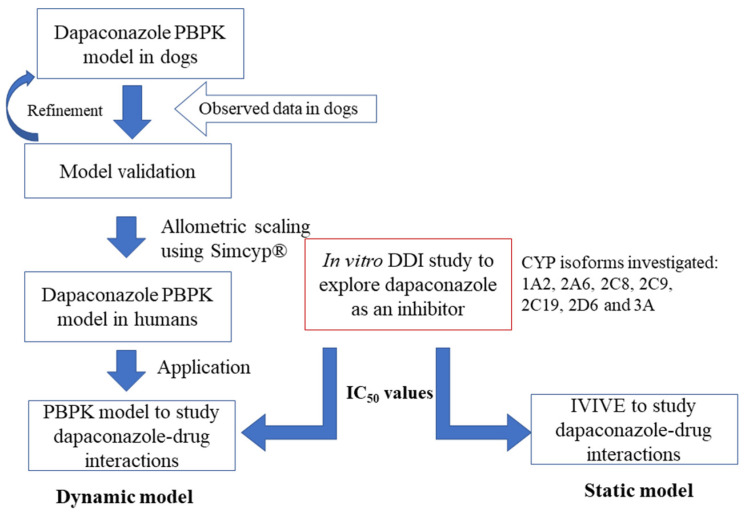
Study workflow.

All modeling was conducted using Simcyp modeling software (v. 20, Certara, Princeton, NJ, US). A PBPK model was constructed to describe the pharmacokinetic profiles of intravenous dapaconazole in dogs, and it was extrapolated to predict drug–drug interactions in humans using a bottom-up approach (Figure 2). All of the input parameters are described in Table 4.

First, a dog PBPK model was developed to describe the observed dapaconazole PK profile from a published study from our research group [7]. The Simcyp minimal PBPK, which considers all organs other than the intestine and liver as a single compartment [36], plus a SAC distribution model were selected. This model better described the bicompartmental distribution profile of dapaconazole [7]. The V_ss_ was predicted using Rodgers and Rowland’s equations (method 2) [15]. A K_p_ scalar was determined to best describe the observed dog data using a sensitivity analysis approach. The final values of V_sac_, k_in_, and k_out_ were determined from the parameter estimation approach selecting the best values to describe the shape of the observed PK profile. For the elimination model development, the in vivo systemic clearance values reported in dogs [7] were employed in a middle-out approach. The intrinsic clearance value from dog liver microsomes obtained from a previous study by our research group [14] was employed to test the role of CYP-mediated hepatic clearance in the systemic elimination of dapaconazole in dogs. The PBPK model was evaluated through a visual comparison of observed in vivo plasma concentration–time profiles with the concentrations predicted in dogs, and the PK parameters within observed/predicted ratios between 0.5-fold to 2-fold were considered acceptable.

The PBPK model developed in dogs considering the in vivo systemic clearance values reported in dogs [7] (middle-out approach) was extrapolated to predict the plasma concentration–time profile in humans and it was used in a prospective prediction of DDI.

The single species allometric scaling tool provided by Simcyp was used to extrapolate the CL and V_ss_ in dogs to humans based on Equation (2):

(2) $${\mathrm{CL}}_{\mathrm{hum}}={\mathrm{bxCL}}_{\mathrm{dog}}{\left(\frac{{\mathrm{BW}}_{\mathrm{hum}}}{{\mathrm{BW}}_{\mathrm{dog}}}\right)}^{a}\;$$

CL_hum_ is the intravenous clearance in humans, CL_dog_ is the intravenous dog clearance, BW_hum_ is the human body weight, BW_dog_ is a dog body weight of 10 kg, and a and b are the allometric coefficient and exponent for dapaconazole, respectively.

(3) $$V_{\mathrm{ss},\mathrm{hum}}=a\;\times {\;V}_{\mathrm{ss},\mathrm{dog}}$$

In Equation (3), V_ss,hum_ is the steady-state volume of distribution in humans, and V_ss,dog_ is the steady-state volume of distribution in the dog.

V_sac_, K_in_, and K_out_ values were kept as estimated for the dog model. The intrinsic clearance from HLM was also tested as the main elimination route. The Simcyp healthy volunteer population was considered, selecting individuals aged from 20 to 50 years old and a gender ratio of 1:1 for all the simulations in humans. The mean demographic parameters were 29.5 years old, 73 kg body weight, and 168.3 cm height. Simulations of 10 trials with 10 subjects were conducted with the dosing regimen of intravenous 20 mg/kg single dose or intravenous 500 mg every 8 h.

### 4.6. Dynamic Model Analysis Using PBPK for DDI Prediction

The potential of dapaconazole to inhibit CYP isoenzymes was predicted using the PBPK model extrapolated to humans. The Ki input values were estimated from the ratio IC_50_/2. Since the IC_50_ experiments were designed considering the substrate concentration equal to Km, the simplification of Ki as IC_50_/2 was considered for a competitive inhibition reaction [37]. For the dynamic DDI model, the trial design included multiple intravenous dapaconazole 500 mg administrations every 8 h for 60 h and starting the protocol at 9 AM on day 1. Each CYP substrate (default substrate model provided by Simcyp) was administered at 9 AM on day 3 in a single oral dose in a fasted state: 150 mg phenacetin (CYP1A2), 0.25 mg repaglinide (CYP2C8), 500 mg tolbutamide (CYP2C9), 200 mg S-mephenytoin (CYP2C19), 20 mg bufuralol (CYP2D6), 5 mg midazolam, and 5 mg nifedipine (CYP3A).

The following Equation (4) was considered for the potential DDI evaluation:

(4) $$\mathrm{AUCR}=\frac{{\mathrm{AUC}}_{\mathrm{with}\;\mathrm{inhibitor}}}{{\mathrm{AUC}}_{\mathrm{without}\;\mathrm{inhibitor}}}$$

where AUCR is the area under the curve ratio, AUC_with inhibitor_ is AUC in the presence of the inhibitor, and AUC_without inhibitor_ is AUC in the absence of the inhibitor.

### 4.7. Static Model Analysis Using IVIVE for DDI Prediction

Initially, the ratio (R1) of intrinsic clearance values of a probe substrate for an enzymatic pathway in the absence and presence of the interacting drug (dapaconazole) for reversible inhibition was calculated according to Equation (5) [13]:

(5) $$R_{1}=1+\left(I_{\max,u}/K_{i,u}\right)$$

where I_max,u_ is the maximal unbound plasma concentration of the interacting drug at steady-state conditions, and K_i,u_ is the unbound inhibition constant determined in vitro.

The static DDI model employed Equation (6) to predict the AUCR:

(6) $$\mathrm{AUCR}=1/\left(\frac{\mathrm{fm}}{\left(1+\frac{\left[I\right]}{\mathrm{Ki}}\right)+\left(1-\mathrm{fm}\right)}\right)$$

where [I] is the simulated _Cmax_, Ki is the inhibition constant, and fm is the fraction metabolized. Ki was corrected to the unbound value using the in vitro unbound fraction (f_u,inc_) of 0.94; Ki = IC_50_/2. The mean dapaconazole C_max_ of 9.5 µM in humans was obtained from simulations of 500 mg every 8 h and corrected by multiplying the ratio of unbound fraction in plasma (f_u_) (0.037) [14] with the blood-to-plasma ratio (R_b_) (6.08 predicted with Simcyp). Nifedipine, midazolam, phenacetin, S-mephenytoin, and bufuralol fm values were adapted from Simcyp; the paclitaxel fm value was extracted from Hua et al. [19]; and the diclofenac fm value was extracted from Siu and Lai [20].

### 4.8. Dapaconazole DDI Results Interpretation

Two recent guidelines provided by the FDA regarding in vitro [13] and clinical [38] DDI studies were considered for interpreting DDI results.

If R_1_ ≥ 1.02, the potential DDI should be investigated further either using mechanistic (static and/or dynamic) models.

The AUCRs obtained with static and dynamic models were evaluated according to the following criteria: AUCR > 1.25 and <2: weak inhibitor; AUCR > 2 and <5: moderate inhibitor; and AUCR ≥ 5: strong inhibitor.

AUCR ≥ 1.25 based on static or dynamic mechanistic models indicates that a clinical DDI study using a sensitive index substrate should be further performed.

## 5. Conclusions

The isoforms CYP2C19, CYP2D6, and CYP3A4 were highly inhibited by dapaconazole, while CYP1A2 was moderately inhibited, CYP2C9 was weakly inhibited, and CYP1A6 was not inhibited when evaluated under in vitro inhibition studies with human liver microsomes. The hybrid intravenous dapaconazole PBPK model developed in dogs described the observed data reasonably well, and it was scaled up to humans. The dynamic (PBPK) and static DDI mechanistic model-based analysis suggest that dapaconazole is a weak inhibitor of CYP1A2 and CYP2C9, a moderate inhibitor of CYP2C8 and CYP2D6, and a strong inhibitor of CYP2C19 and CYP3A, considering a clinical scenario. The results presented may be a useful guide for future in vivo and clinical dapaconazole studies.

## Figures and Tables

**Table 1:** Conditions of the in vitro CYP450 metabolism inhibition assay.

CYP450 Isoform	Substrate	Substrate Concentration (µM)	Marker	Inhibitor	Inhibitor Concentration (µM)	Microsome Concentration (mg/mL)	Incubation Time (min)	Internal Standard (Solvent)
**1A2**	Phenacetin	12.03	Acetominofen	Furafylline	0.1–2.3	0.3	30	Sulindac (MTBE)
**2A6**	Coumarin	2.3	7-Hydroxycoumarin	Tranylcypromine	0.1–2.0	0.3	30	Dextrorphan (EA)
**2B6**	Bupropion	81.7	Hydroxybupropion	Clopidogrel	0.01–0.05	0.1	10	Sulindac (ACN)
**2C8**	Paclitaxel	10.0	6α-Hydroxypaclitaxel	Quercetin	1–5	0.4	20	Dexthorphan (EA)
**2C9**	Diclofenac	4.04	4′-Hydroxydiclofenac	Sulfaphenazole	0.1–2.0	0.1	10	Sulindac (CF ^1^)
**2C19**	S-mephenytoin	57.2	4′-Hydroxymephenytoin	Tranylcypromine	5–45	0.2	40	Dextrorphan (EA)
**2D6**	Bufuralol	5.4	1′-Hydroxybufuralol	Quinidine	0.001–0.3	0.25	30	Dextrorphan (EA ^2^)
**3A**	Midazolam	2.27	1-Hydroxymidazolam	Ketoconazole	0.01–0.05	0.1	5	Diazepam (EA)
**3A**	Nifedipine	7.0	Dehydronifedipine	Ketoconazole	0.01–0.05	0.15	15	Diazepam (EA)

Modifier: ^1^ HCl 0.5 M; ^2^ NaOH 1.25 µM. MTBE, methyl tert—butyl ether; EA, ethyl acetate; ACN, acetonitrile; CF, chloroform.

**Table 2:** LC-MS/MS conditions.

CYP Isoform	Marker	MRM Transitions	CE (Volts)	CXP (Volts)
**1A2**	Acetominofen	152.11 > 109.90 152.11 > 65.20	23 43	08 04
**2A6**	7-Hydroxycoumarin	162.99 > 107.00 162.99 > 77.10	31 47	08 06
**2B6**	Hydroxybupropion	256.22 > 238.00 256.22 > 238.00	17 35	14 12
**2C8**	6α-Hydroxypaclitaxel	870.42 > 139.00 870.42 > 104.90	21 77	08 10
**2C9**	4′-Hydroxydiclofenac	312.02 > 231.10 312.02 > 231.10	27 43	14 20
**2C19**	4′-Hydroxymephenytoin	235.11 > 150.10 235.11 > 141.00	25 15	12 10
**2D6**	1′-Hydroxybufuralol	278.25 > 186.00 278.25 > 159.00	25 33	26 12
**3A ^1^**	1-Hydroxymidazolam	342.06 > 234.00 342.06 > 108.90	31 45	14 08
**3A ^2^**	Dehydronifedipine	345.00 > 283.90 345.00 > 267.80	35 26	10 08
**IS**	Diazepam	285.12 > 154.10 285.12 > 193.00	37 43	10 16
**IS**	Sulindac	357.14 > 233.10 357.14 > 233.10	59 47	16 14
**IS**	Dextrorphan	258.30 > 157.10 258.30 > 199.10	49 37	10 18

^1^ Midazolam as substrate; ^2^ Nifedipine as substrate; CE, collision energy; CXP, collision cell exit potential; and IS, internal standard.

**Table 3:** IC_50_ values obtained from the in vitro CYP450 metabolism inhibition assays.

CYP Isoform	IC_50_ (µM) Dapaconazole	IC_50_ (µM) Positive Control
**1A2**	3.682 (0.1295)	0.5847 (0.08698)—Furafylline
**2A6**	20.7 (0.0561)	0.7994 (0.08698)—Tranylcypromine
**2C8**	104.1 (0.4935)	0.6221 (0.5273)—Quercetin
**2C9**	0.2186 (0.1047)	0.4467 (0.3811)—Sulfaphenazole
**2C19**	0.05297 (0.01904)	0.4467 (0.3811)—Tranylcypromine
**2D6**	0.8675 (0.2102)	0.03712 (0.07987)—Quinidine
**3A ^1^**	0.007693 (0.001267)	0.003445 (1.161)—Ketoconazole
**3A ^2^**	0.03032 (0.05029)	0.003667 (0.3481)—Ketoconazole

^1^ Midazolam as substrate; and ^2^ Nifedipine as substrate.

**Table 4:** Input parameters for the dapaconazole PBPK model in dogs and humans.

	Dog Model		Human Model	
Parameters	Value	Reference	Value	Reference
**Physical chemistry**				
Molecular weight (g/mol)	415.2	Drugbank	415.2	Drugbank
log P	5.63	Drugbank	5.63	Drugbank
pKa (monoprotic base)	6.77	Drugbank	6.77	Drugbank
Unbound fraction	0.037	Antunes et al. [14]	0.077	Antunes et al. [14]
Blood/Plasma	1 *	Assumed	6.08	Simcyp predicted
**Distribution**	Minimal + SAC model		Minimal+ SAC model	
V_ss_ (L/kg)	6.359	Predicted Method 2	6.35	Simcyp Allometry (simple allometry)
V_sac_ (L/kg)	3.883	Best fit	3.883	Allometry
K_in_/K_out_ (1/h)	0.0262/0.01582	Best fit	0.0262/0.01582	Allometry
K_p_	0.01	Best fit	0.01	Allometry
**Elimination**				
CL IV (L/h)	According to IV dose simulated	Palo et al. [7]	35.5	Simcyp Allometry (simple allometry)
CL int. mic. µL/min/mg	258	Antunes et al. [14]	118.5	Antunes et al. [14]
f_u,inc_	0.97	Antunes et al. [14]	0.94	Antunes et al. [14]

* This value was assumed 1 as a default value provided by Simcyp due to the lack of experimental data. V_ss_, volume of distribution at steady-state conditions; SAC, single adjusting compartment; V_sac_, apparent volume of SAC; K_in_/K_out_, rate constant from systemic compartment to SAC/rate constant from SAC compartment to the systemic compartment; K_p_, tissue-to-plasma partition coefficient; CL IV, intravenous clearance; CL int. mic., intrinsic clearance obtained from liver microsomes; and f_u,inc_, unbound fraction.

**Table 5:** Prediction performance of the dapaconazole PBPK model in dogs (model qualification).

Intravenous Dose	1 mg/kg			
Parameters	AUC_0-t_ (ng/mL.h)	C_max_ (ng/mL)	CL (mL/min)	t_1/2_ Terminal (h)
**Predicted**	306.7	404.7	543.5	1.9
**Observed ^1^**	255.0	373.2	700.0	2.1
**Observed/predicted ratio**	0.83	0.92	1.29	1.08
	**2 mg/kg**			
**Predicted**	613.3	809.3	543.5	1.9
**Observed ^1^**	779.9	1444.7	591.7	2.5
**Observed/predicted ratio**	1.27	1.78	1.09	1.29
	**20 mg/kg**			
**Predicted**	7331.2	8097.3	454.7	2.2
**Observed ^1^**	4780.1	4708.3	700.0	2.3
**Observed/predicted ratio**	0.65	0.58	1.54	1.04

^1^ Data from Palo et al. [7]. AUC_0-t_, the area under the curve zero to last time of sample collected; C_max_, maximum plasma concentration; and CL, clearance; and t_1/2_ terminal, terminal half-life.

**Table 6:** Evaluation of the potential of dapaconazole as a CYP inhibitor through the predicted area under the curve ratios using a static and dynamic model.

CYP.	Substrate	[I](µM)	Ki (µM)	R1	fm	Static AUCR	Dynamic AUCR	FDA Classification [13]
**1A2**	Phenacetin	9.5	1.84	1.20	0.71	1.86	1.17	Weak
**2C8**	Paclitaxel	9.5	52.05	1.01	0.5	3.00	1.46 ^1^	Moderate
**2C9**	Diclofenac	9.5	0.11	4.43	0.87	1.95	1.38 ^2^	Weak
**2C19**	S-Mephenytoin	9.5	0.03	15.14	0.89	3.86	5.36	Strong
**2D6**	Bufuralol	9.5	0.43	1.86	0.66	2.31	1.51	Moderate
**3A4**	Midazolam	9.5	0.004	98.26	0.88	19.45	5.14	Strong
**3A4**	Nifedipine	9.5	0.02	25.70	0.96	5.31	4.05	Strong

^1^ AUCR dynamic study was performed in Simcyp using repaglinide as a CYP2C8 substrate. ^2^ AUCR dynamic study was performed in Simcyp using tolbutamide as a CYP2C9 substrate. Abbreviations: R1, ratio of intrinsic clearance values of a probe substrate for an enzymatic pathway in the absence and the presence of dapaconazole; CYP, cytochrome P450; fm, fraction metabolized; [I], inhibitor concentration that is the total plasma maximum concentration (C_max_); Ki, inhibition constant; and AUCR, area under the curve ratio between AUC with inhibitor and AUC without inhibitor. Notes: Ki was corrected to the unbound value using the in vitro unbound fraction (f_u,inc_) of 0.94 Ki = IC_50_/2; the mean C_max_ of 9.5 µM in humans was obtained from simulations of 500 mg every 8 h and corrected by multiplying the ratio of unbound fraction in plasma (fu) with the blood-to-plasma ratio (Rb); the fm of nifedipine, midazolam, phenacetin, S-mephenytoin, and bufuralol were adapted from Simcyp. Paclitaxel was extracted from Hua et al. [19]; and diclofenac from Siu and Lai [20]. AUCR > 1.25 and <2: weak inhibitor; AUCR > 2 and <5: moderate inhibitor; and AUCR ≥ 5: strong inhibitor.

## Data Availability

Data is contained within the article.
